# Supplementary material for: Identification of photoperiod-induced specific miRNAs in the adrenal glands of Sunite sheep (Ovis aries)
Source: Front Vet Sci. 2022 Jul 22;9:888207. doi: 10.3389/fvets.2022.888207 (PMC9354845; doi:10.3389/fvets.2022.888207)
Supplement: Supplementary file 1 [file Data_Sheet_1.ZIP › Supplementary file/miRDeep2 file.zip!/miRDeep2 file/Novel_Structure/SPLP42A1/Novel_394.pdf]

Provisional ID	:	NC_019458.2_192
Score total	:	0
Score for star read(s)	:	-1.3
Score for read counts	:	-2.3
Score for mfe	:	2
Score for randfold	:	1.6
Score for cons. seed	:	
Total read count	:	7
Mature read count	:	7
Loop read count	:	0
Star read count	:	0

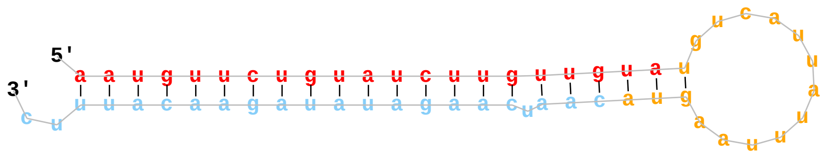

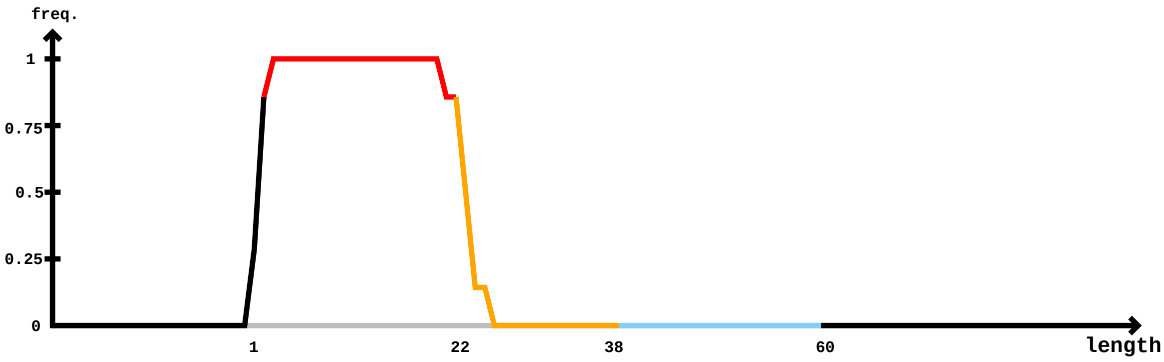

Mature Star

```
5'- ggaaaccuggggguaauggaaauguucuguaucuuguuguaugucauuauuuaaguacaaucaagauauagaacauuuccauuacacucaagcuuaauuaaauggauauau   -3'    exp
    .......((((.((((((((((((((((((((((((((((((...........)))))).))))))))))))))))))))))))).)))).....................   reads     mm        sample
    ...................aaauguucuguaucuuguugua.......................................................................   2         0         seq
    ....................aauguucuguaucuuguug.........................................................................   1         0         seq
    ....................aauguucuguaucuuguugua.......................................................................   3         0         seq
    .....................auguucuguaucuuguuguaugu....................................................................   1         0         seq
```
